# Immunoreactivity of humanized single-chain variable fragment against its functional epitope on domain 1 of CD147

**DOI:** 10.1038/s41598-022-10657-3

**Published:** 2022-04-25

**Authors:** Nutjeera Intasai, Kuntalee Rangnoi, Montarop Yamabhai, Thanathat Pamonsupornwichit, Weeraya Thongkum, Umpa Yasamut, Koollawat Chupradit, Nuchjira Takheaw, Piyarat Nimmanpipug, Chatchai Tayapiwatana

**Affiliations:** 1 Division of Clinical Microscopy, Department of Medical Technology, Faculty of Associated Medical Sciences, Chiang Mai University, Chiang Mai, Thailand; 2 Center of Biomolecular Therapy and Diagnostic, Faculty of Associated Medical Sciences, Chiang Mai University, Chiang Mai, Thailand; 3 Center of Innovative Immunodiagnostic Development, Department of Medical Technology, Faculty of Associated Medical Sciences, Chiang Mai University, Chiang Mai, Thailand; 4 Molecular Biotechnology Laboratory, School of Biotechnology, Institute of Agricultural Technology, Suranaree University of Technology, Nakhon Ratchasima, Thailand; 5 Division of Clinical Immunology, Department of Medical Technology, Faculty of Associated Medical Sciences, Chiang Mai University, Chiang Mai, Thailand; 6 Siriraj Center for Regenerative Medicine, Faculty of Medicine Siriraj Hospital, Mahidol University, Bangkok, Thailand; 7 Biomedical Technology Research Center, National Center for Genetic Engineering and Biotechnology, National Science and Technology Development Agency at the Faculty of Associated Medical Sciences, Chiang Mai University, Chiang Mai, Thailand; 8 Department of Chemistry, Faculty of Science, Chiang Mai University, Chiang Mai, Thailand

**Keywords:** Biotechnology, Cancer, Drug discovery, Immunology

## Abstract

Domain 1 of CD147 participates in matrix metalloproteinase (MMP) production and is a candidate for targeted therapy to prevent cancer invasion and metastasis. A functional mouse anti-CD147 monoclonal antibody, M6-1B9, was found to recognize domain 1 of CD147, and its respective mouse single-chain variable fragment (ScFvM61B9) was subsequently generated. The EDLGS epitope candidate for M6-1B9 was identified using the phage display peptide technique in this study. For future clinical applications, humanized ScFv specific to domain 1 of CD147 (HuScFvM61B9) was partially adopted from the hypervariable sequences of parental mouse ScFvM61B9 and grafted onto suitable human immunoglobulin frameworks. Molecular modelling and simulation were performed in silico to generate the conformational structure of HuScFvM61B9. These results elucidated the amino acid residues that contributed to the interactions between CDRs and the epitope motif. The expressed HuScFvM61B9 specifically interacted with CD147 at the same epitope as the original mAb, M6-1B9, and retained immunoreactivity against CD147 in SupT1 cells. The reactivity of HuScFvM61B9 was confirmed using CD147 knockout Jurkat cells. In addition, the inhibitory effect of HuScFvM61B9 on OKT3-induced T-cell proliferation as M6-1B9 mAb was preserved. As domain 1 is responsible for cancer invasion and metastasis, HuScFvM61B9 would be a candidate for cancer targeted therapy in the future.

## Introduction

CD147, a transmembrane glycoprotein in the immunoglobulin superfamily, is widely expressed in various cell types and tissues and exerts a diverse range of physiological and pathological processes^1^. CD147 is overexpressed in cancer and plays a fundamental role in regulating the tumor microenvironment and cancer progression by several mechanisms, including proliferation, invasion, metastasis, angiogenesis, glycolysis, and chemoresistance^2,3^. CD147 was shown to stimulate the production of MMP-1, MMP-2, MMP-3 in melanoma^4^, MMP-9 in breast cancer^5^, MMP-11 in colorectal cancer^6,7^ and MMP-2 and MMP-9 in hepatocellular carcinoma^8–10^. CD147 also plays a crucial role in T cell activation and proliferation; however, CD147 seems to display different functional activities at different phases of T cell responses^11–14^.

Domain 1 (IgC2 domain) of CD147 is important in matrix metalloproteinase (MMP) production, which leads to the degradation of the basement membrane and extracellular matrix and promotes tumor proliferation, invasion, and metastasis^15^. The disruption of CD147 dimerization can prevent cancer cell invasion and metastasis^8^. In comparison to the strengths of other therapeutic antibodies, immunotargeting of the CD147 functional domains is considered a promising candidate for cancer immunotherapy in preventing cancer invasion and metastasis^16–24^. A therapeutic mouse anti-CD147 monoclonal antibody (mAb), HAb18, recognizes the residue ^39^LTCSLNDSATEV^50^ in domain 1 of CD147 and exerts an anti-metastatic activity in hepatocellular carcinoma through the reduction of MMPs^16,17^.

The functional epitope of M6-1B9, a mouse anti-CD147 mAb, was formerly characterized as residing on domain 1^25,26^. Subsequently, mouse single-chain fragment variable of M6-1B9 (ScFvM61B9) was generated from the cDNA of hybridoma clone M6-1B9 to be expressed as soluble ScFvM61B9 and ScFvM61B9 intrabody forms^27^. Soluble ScFvM61B9 inhibited T-cell proliferation after stimulation with an anti-CD3 mAb^28^. ScFvM61B9 activity has not yet been studied in solid tumors. However, the ScFvM61B9 intrabody was previously found to inhibit the cell surface expression of CD147 and decreased the aggressiveness of various cancers via specific mechanisms^28–32^. Although the ScFvM61B9 intrabody did not affect either MMP-2 or MMP-9 activities in the cervical cancer HeLa cell line, it suppressed urokinase-type plasminogen activator (uPA) and inhibited HeLa cell metastasis^28,31^. In addition, the ScFvM61B9 intrabody affected the α3β1-integrin and MCT-1 function and suppressed the progressive phenotype in the colorectal cancer Caco-2 cell line^29^. Moreover, the ScFvM61B9 intrabody promoted intracellular acidosis and induced apoptosis in the Caco-2 cell line^30^. Correspondingly, immunotargeting of CD147 domain 1 is feasible for cancer immunotherapy.

This present study aimed to verify the epitope on CD147 extracellular domain recognized by M6-1B9 mAb. In addition, the peptide sequences of heavy and light chain frameworks of its mouse single chain variable (scFv; ScFvM61B9) were replaced with human frameworks (HuScFvM61B9) and the biological activity of this humanized scFv was assessed for future clinical applications.

## Results

### Identification of M6-1B9 monoclonal antibody epitope through biopanning of phage display combinatorial peptide library

**Figure 1:**
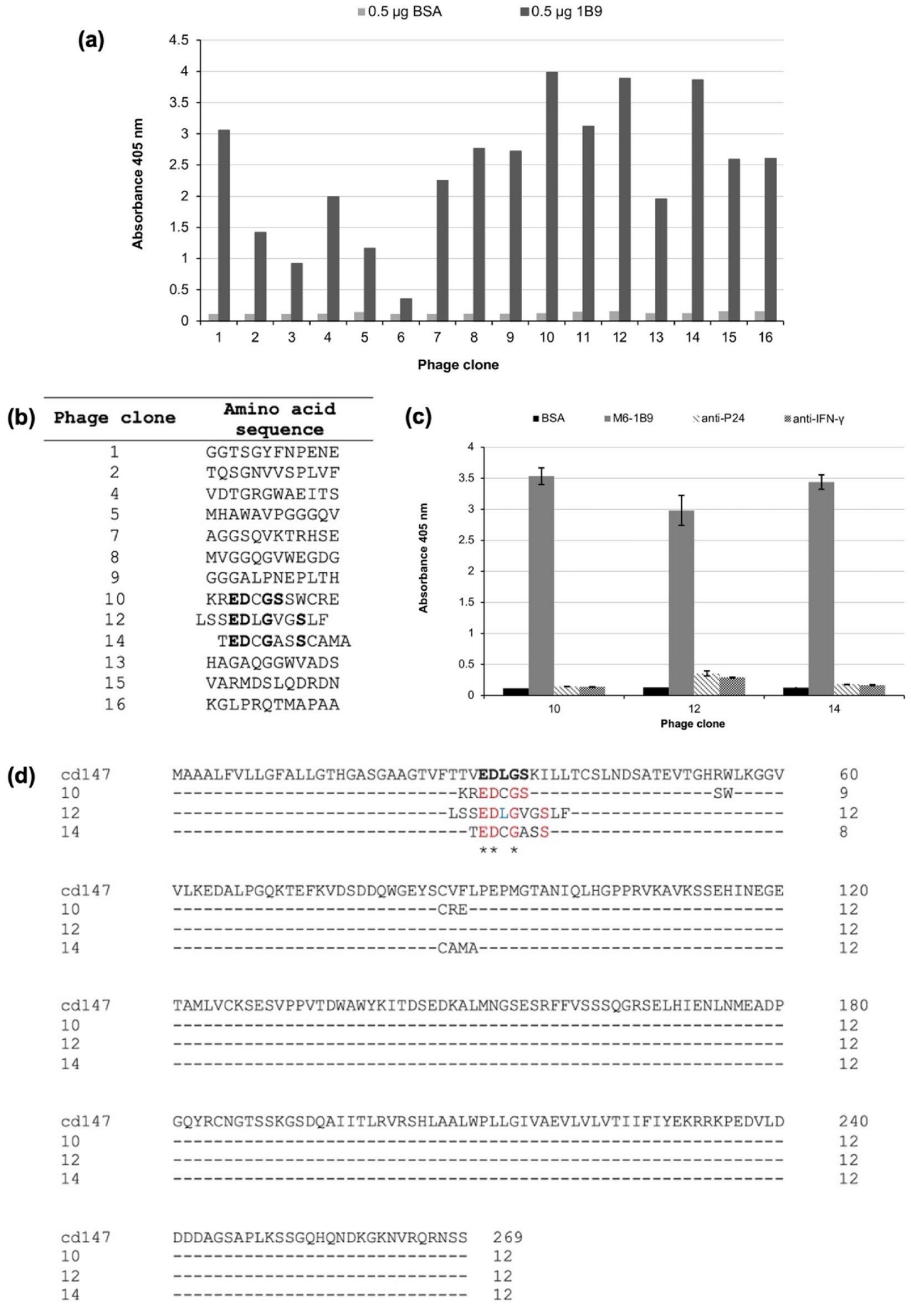
Binding of phage clones to M6-1B9 mAb and the predicted motif, EDxGS. (**a**) Binding activity of selected phage clones to M6-1B9 mAb via phage peptide ELISA. The ELISA wells were coated with 0.5 µg of M6-1B9 mAb and 0.5 µg BSA as a negative control. Bound phage was detected with an HRP-conjugated anti-M13 antibody. The absorbance at 405 nm was measured using a microtiter plate reader. (**b**) Amino acid sequences of the 12-mer peptides from selected phage clones. The amino acids of each CDR interacting with the epitope on domain 1 of CD147 are depicted in red letters. (**c**) Confirmation of binding activity of positive phage peptides via ELISA. Three identified mimotope peptides possessing a consensus motif were tested for the binding against M6-1B9, anti-P24, and anti-IFN γ mAbs. Bound phage was detected with an HRP-conjugated anti-M13 antibody. Triplicate measurements of absorbance values at 405 nm with standard deviations were reported. (**d**) Amino acid sequence alignment of selected phage clones and human CD147. The predict motif EDxGS is shown in red letters, and is correlated to the amino acid on CD147 (bold). The CD147 human sequence is from GeneBank (AB085790.1).

After three rounds of bio-panning, 16 phage clones were randomly chosen to identify the bona fide binding partner to M6-1B9 mAb via phage enzyme-linked immunosorbent assay (ELISA) (Fig. 1a). Fourteen clones that showed specific binding to M6-1B9 mAb were selected to determine the DNA and amino acid sequences. One phage clone (11) showed no peptide sequences. The deduced peptide sequences are presented in Fig. 1b. Among them, three clones (10, 12, and 14) shared the EDxGS motif (x is any amino acid residue). The specific binding activity of these clones to M6-1B9 mAb was confirmed again with an isotype control by ELISA, as shown in Fig. 1c. Phages 10, 12, and 14 could bind specifically to M6-1B9 mAb but not to the anti-P24 and anti-IFN-γ mAbs. The amino acid sequences of these three clones were aligned with the human CD147 sequence using Clustal Omega. Alignment revealed that all three clones shared less than three continuous amino acid residues identical to the sequence of human CD147 registered in GenBank, which indicated that these peptides were likely the mimotope of CD147 (Fig. 1d). Because the EDLGS motif is not present in mouse CD147, the retrieved phage peptide demonstrates a strong tendency to be an antigenic determinant.

### Humanization of mouse ScFvM61B9

**Figure 2:**
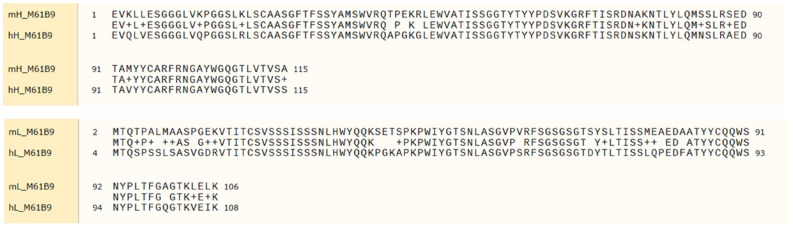
Alignment result of mouse ScFvM61B9 (mouse light chain; mL and mouse heavy chain; mH) against HuScFvM61B9 (human light chain; hL and human heavy chain; hH).

The original peptide sequences of the heavy and light chain frameworks of mouse ScFvM61B9 were automatically substituted with human frameworks via BioLuminate analysis. The alignment of the amino acid sequences of parental ScFvM61B9 versus HuScFvM61B9 is shown in Fig. 2. The T20 humanness score improved from 92.3 to 100.05 and 60.9 to 82.54 for heavy and light chains, respectively. The human germlines of HuScFvM61B9 are IGHV3 and IGKV1.

### Molecular model of HuScFvM61B9 against CD147 domain 1

**Figure 3:**
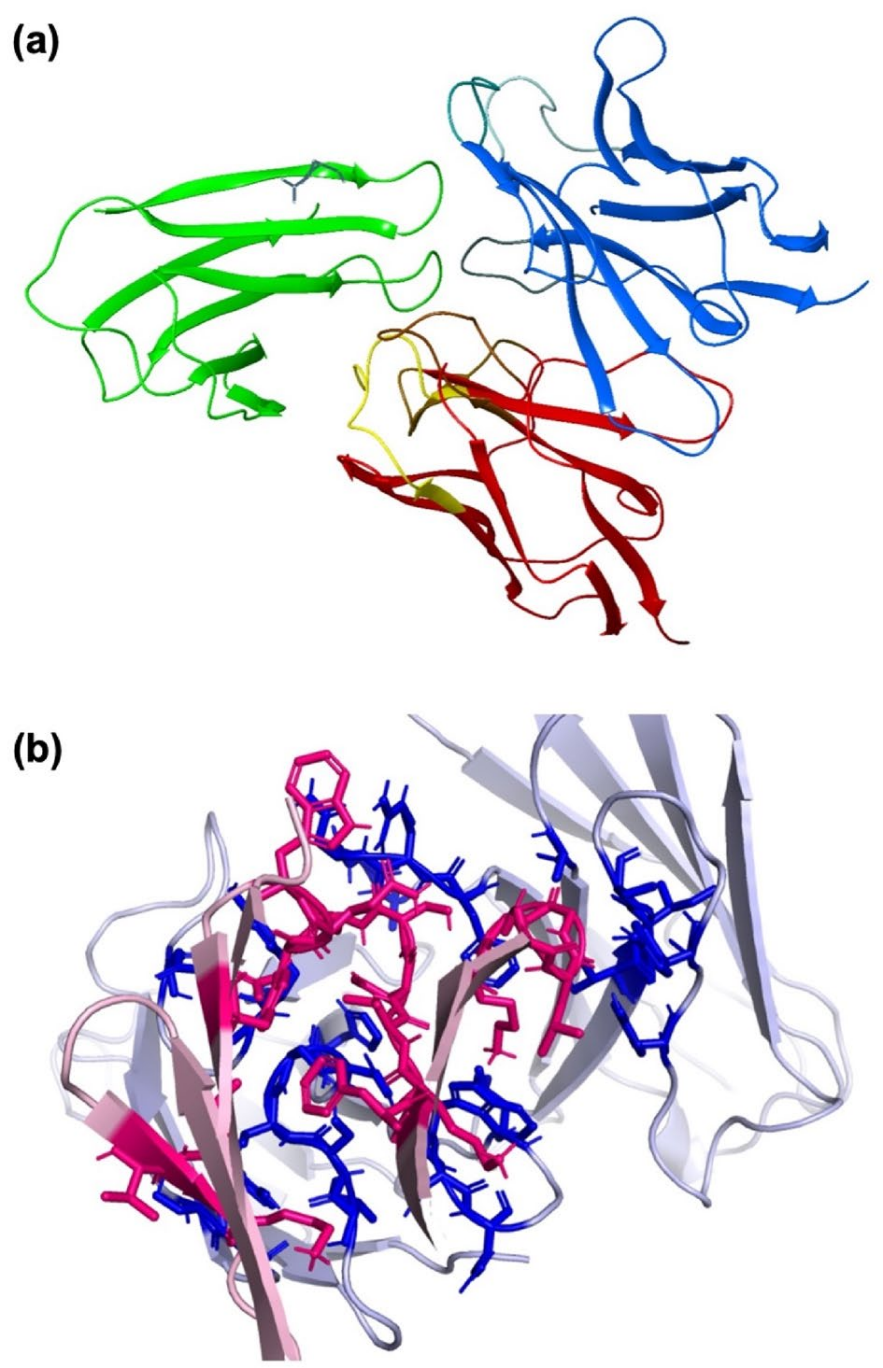
Model and amino acid residues participating in the interaction of HuScFvM61B9 against domain 1 of CD147. (**a**) Automatic masking of non-CDR regions of the antibody mode in ClusPro 2.0 is applied to predict the interaction coordinate of HuScFvM61B9 against domain 1 of CD147 conformational structures generated from BioLuminate 4.0. The H-chain and L-chain variable domains are shown as blue and red ribbons, respectively. The domain 1 target is depicted as a green ribbon. (**b**) The Prodigy Web server was used to elucidate the molecular interaction of the selected complex from the ClusPro 2.0 output. Based on the Pymol code automatically generated by Prodigy, the crucial side-chain residues on CDRs of HuScFvM61B9 and domain 1 (consisting of the EDLGS epitope) at the binding interface are labeled in blue and red, respectively. The predicted binding affinity (K_D_) is 1.60 × 10^–8^.

Ten candidate complexes were obtained from the ClusPro 2.0 Web server. The interactive residues on the predicted complexes were analyzed using the Prodigy Web server^33^. The most likely pose illustrating the interactive epitope residues correlating with the amino acid sequence identified using the phage-display peptide was verified (Fig. 3a). Pymol software was used to visualize the residues found on the contact surface using the Pymol code generated from Prodigy (Fig. 3b).

Amino acid residue contributions and interaction types were examined to understand the intermolecular interactions that can improve discovery. We estimated a possible intermolecular residue interaction based on the atomic coordinates from the selected candidate. We identified hydrogen bonds of the D-H…A type (donor atom D and acceptor atom A) using the following distance and angle criteria: *d*(D…A) ≤ 3.5 Å, *θ*(D-H…A) ≥ 90°. Atom-π, π-π, and hydrophobic *van der Waals* interactions were analyzed using the centroid–centroid and between heavy atoms criteria with a maximum distance of 6.0 Å.

### Analysis of generated HuScFvM61B9 structure

**Figure 4:**
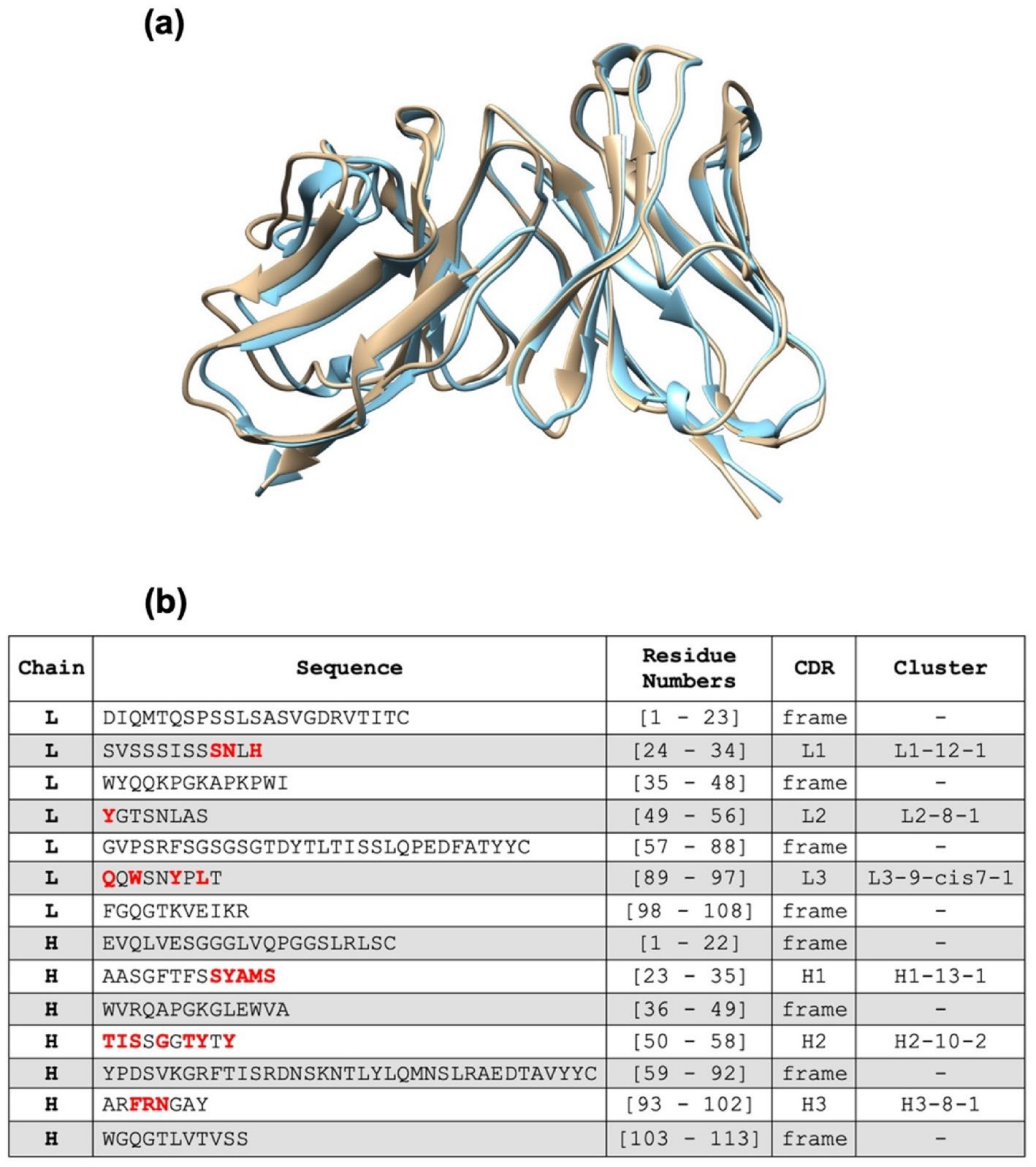
Structural comparison of parental ScFvM61B9 (gold ribbon) versus HuScFvM61B9 (blue ribbon) and identification and clustering of HuScFvM61B9 CDRs. The conformational structures were obtained from the BioLuminate 4.0 antibody humanization process. The calculated RMSD between 108 pruned atom pairs is 0.758 Å (across all 115 pairs: 1.004) as per the MatchMaker function in the UCSF Chimera 1.15 software with the Needleman-Wunsch algorithm/BLOSUM-62. (**b**) The amino acids of each CDR interacting with the epitope on domain 1 of CD147 are depicted in red letters.

The original mouse ScFvM61B9 and the predicted HuScFvM61B9 structures using BioLuminate were compared (Fig. 4a). The analyzed root-mean-square deviation (RMSD) was 0.758 Å. The CDRs of HuScFvM61B9 were identified using PyIgClassify on the HuScFvM61B9 structure (Fig. 4b). The most likely complex of HuScFvM61B9 and domain 1 of CD147 was selected from ClusPro 2.0 based on the epitope; that is, EDLGS was retrieved from the phage peptide analysis. The molecular interaction of HuScFvM61B9 against the epitope residing in domain 1 of CD147 was analyzed using Prodigy.

### Atomic-level intermolecular interactions in the HuScFvM61B9-CD147 binding structure

**Figure 5:**
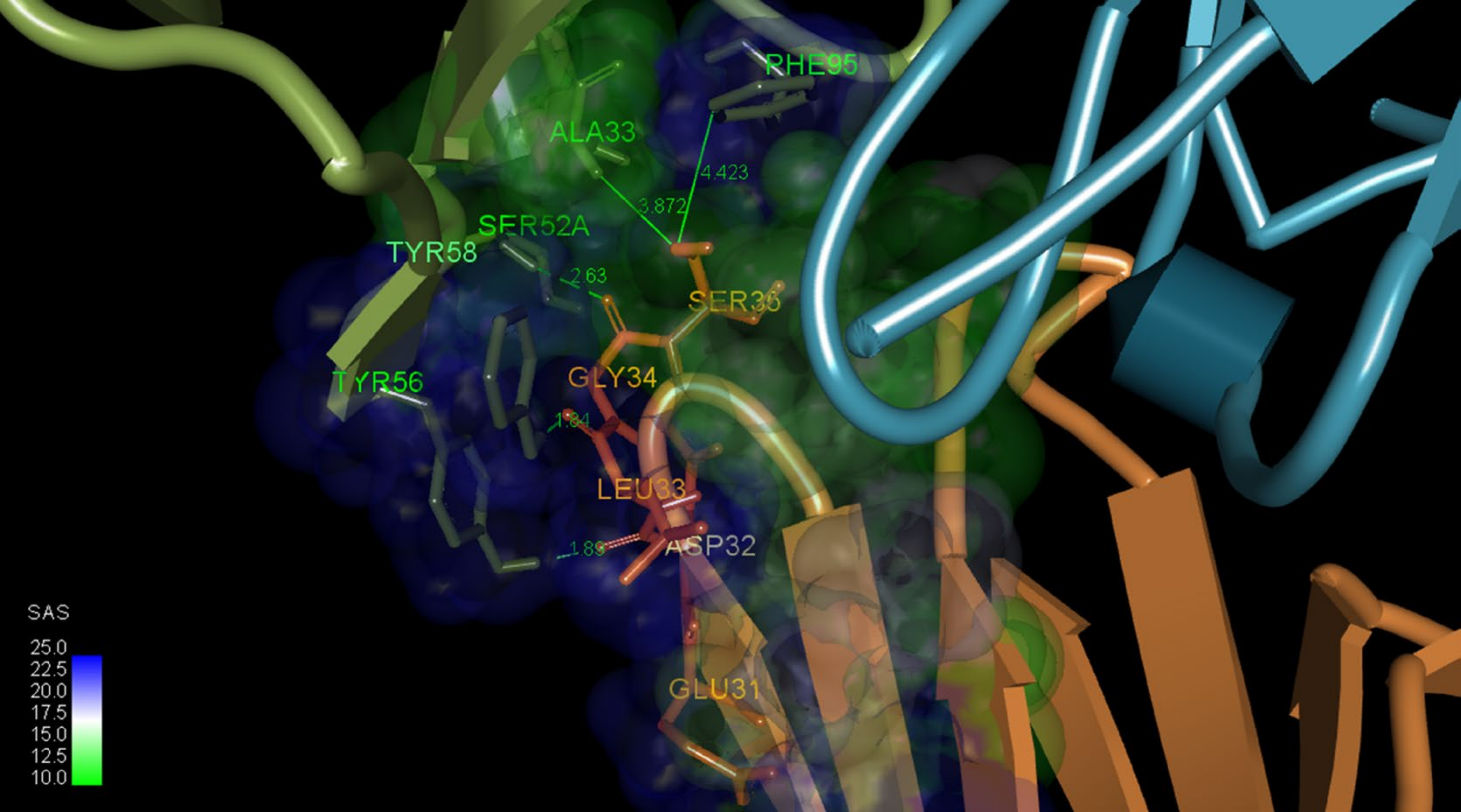
Atomic-level observation of amino acid residues participating in the HuScFvM61B9-CD147 predicted binding structure. Key residues on CDRs of HuScFvM61B9 and domain 1 of CD147 at the binding surface are highlighted in green and orange, respectively.

This atomistic picture of the structural assembly was in accordance with the epitope mapping analysis results. The complex structure suggested that hydrogen bonding and *van der Waals* interactions are essential in this protein–protein association. The crucial side-chain residues on CDRs of HuScFvM61B9 and domain 1 (composed of EDLGS epitope) at the binding surface are labeled in Fig. 5. From the complex, S52, Y56, and Y58 in HuScFvM61B9 CDR H2 formed hydrogen bonds with domain 1 of CD147. For HuScFvM61B9, CDR H1, H3, A33, and F95 interacted with domain 1 of CD147 at a longer distance via* van der Waals* interactions.

### Expression of recombinant protein HuScFvM61B9

**Figure 6:**
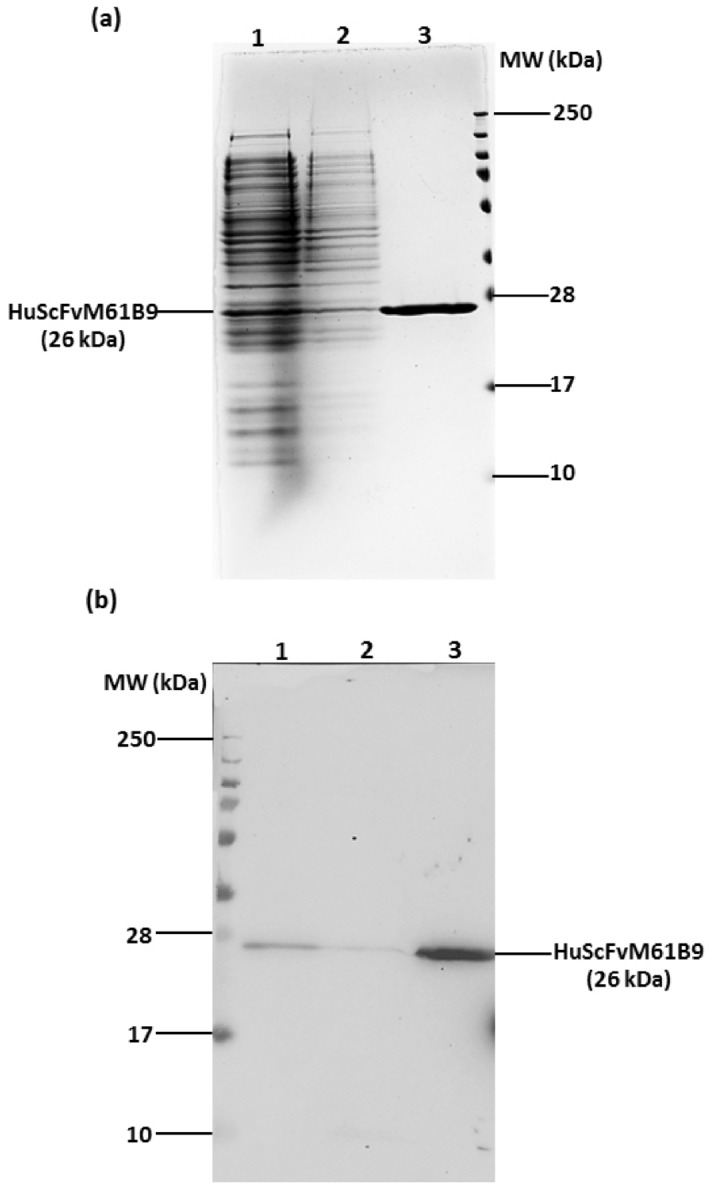
Detection of purified HuScFvM61B9 via SDS-PAGE and Western blot analysis. (**a**) The crude proteins extracted from bacteria and the purified recombinant protein were subjected to 15% SDS-PAGE analysis. Crude lysate proteins (lane 1), crude pellet proteins (lane 2), and purified HuScFvM61B9 (lane 3) are shown. The protein bands were visualized through PAGE Blue staining. (**b**) Proteins were separated by SDS-PAGE, transferred onto a PVDF membrane, and subsequently probed with an HRP-conjugated anti-His-tag antibody (dilution 1:3000). The reaction was developed using a chemiluminescent substrate detection system. Crude lysate proteins (lane 1), crude pellet proteins (lane 2), and purified HuScFvM61B9 (lane 3) are shown.

The HuScFvM61B9 protein was expressed in *Escherichia coli* (*E. coli*) Origami B (DE3) and purified via affinity chromatography. The results revealed that the purified HuScFvM61B9 protein containing a His6x tag was successfully produced. The recombinant protein at approximately 26 kDa was detected via SDS-PAGE (Fig. 6a) and Western immunoblotting (Fig. 6b).

### Binding affinity of HuScFvM61B9 in comparison to M6-1B9 mAb

**Figure 7:**
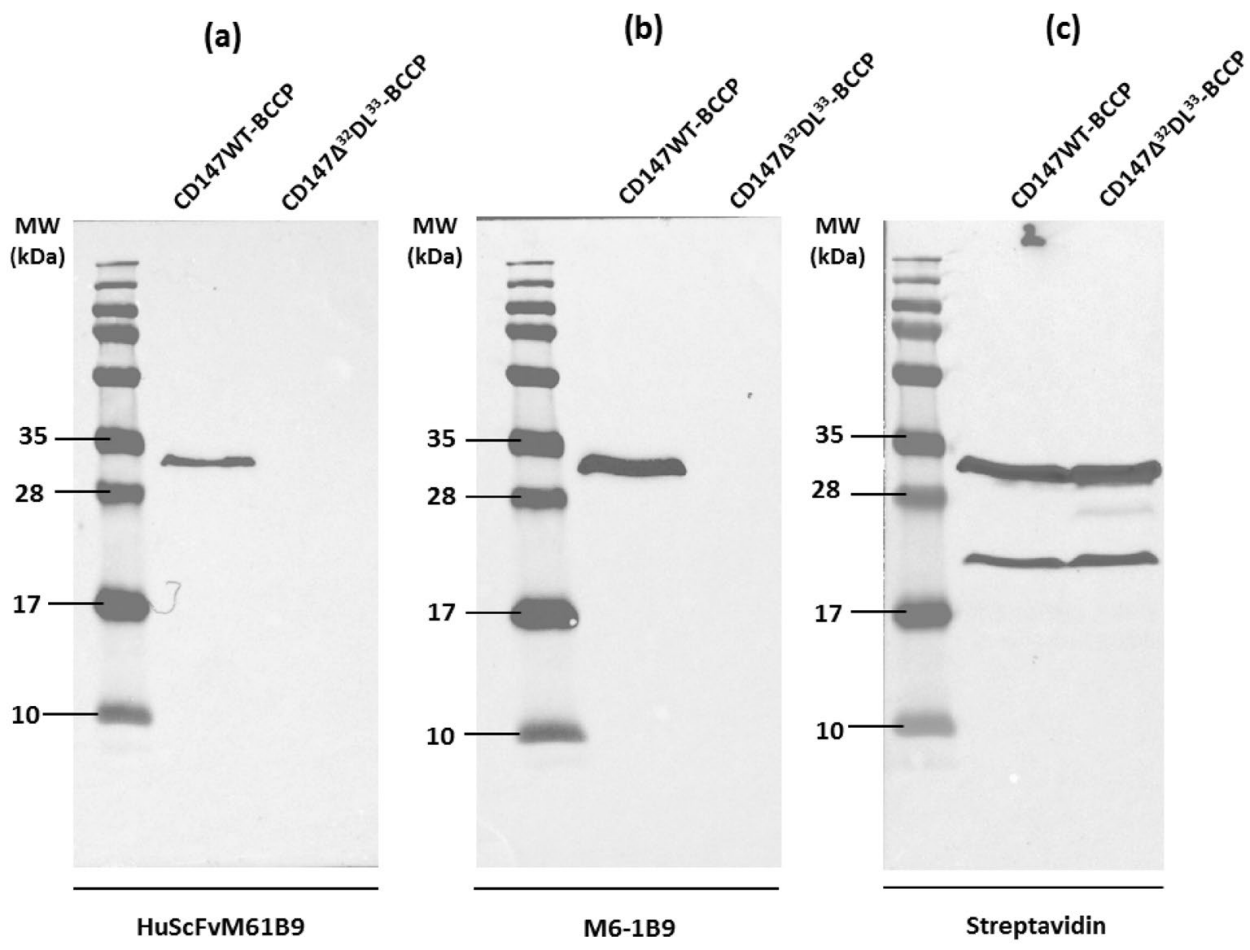
Interaction of HuScFvM61B9 against the wild-type CD147 and mutant CD147. The crude lysates from *E. coli* strain Origami B containing wild-type CD147-BCCP (CD147WT-BCCP) and mutant CD147-BCCP (CD147Δ^32^DL^33^-BCCP) were subjected to 15% SDS-PAGE analysis, transferred onto a PVDF membrane, and subsequently probed with HuScFvM61B9 (**a**) and anti-CD147 (M6-1B9) mAb (**b**). The immunoreactivity was subsequently detected using HRP-conjugated anti-His-tag mAb (**a**), HRP-conjugated rabbit anti-mouse Igs (**b**) and HRP-conjugated Streptavidin (**c**). The reaction was developed using a chemiluminescent substrate detection system.

To compare the binding affinity between HuScFvM61B9 with its parental mouse mAb, M6-1B9, to human CD147, biolayer interferometry was performed using Streptavidin biosensor tip (Sartorius FortéBio) and human CD147-BCCP. The Western blot analysis of the produced CD147-BCCP was shown (Fig. 7). The K_D_ at 7.97 ± 0.53 × 10^–8^ for HuScFvM61B9 and 3.11 ± 1.28 × 10^–9^ for M6-1B9 was assessed based on the association and dissociation constants using the BLitzPro 1 program.

### Interaction of HuScFvM61B9 against wild-type CD147 and mutant CD147

Wild-type CD147 (CD147WT-BCCP) and mutant CD147 (CD147Δ^32^DL^33^-BCCP) were expressed in *E. coli* strain Origami B to assess the specificity of HuScFvM61B9 by Western immunoblotting. The HuScFvM61B9 was specifically reacted to the band at ~ 35 kDa of CD147WT-BCCP only while the band at ~ 35 kDa of CD147Δ^32^DL^33^-BCCP was absent (Fig. 7a). This reactive band was concordant with the interaction against its parental M6-1B9 mAb (Fig. 7b). In addition, the equal band intensity at ~ 35 kDa from CD147WT-BCCP and CD147Δ^32^DL^33^-BCCP was demonstrated using the HRP-conjugated Streptavidin probe (Fig. 7c).

### Determination of HuScFvM61B9 binding activity

**Figure 8:**
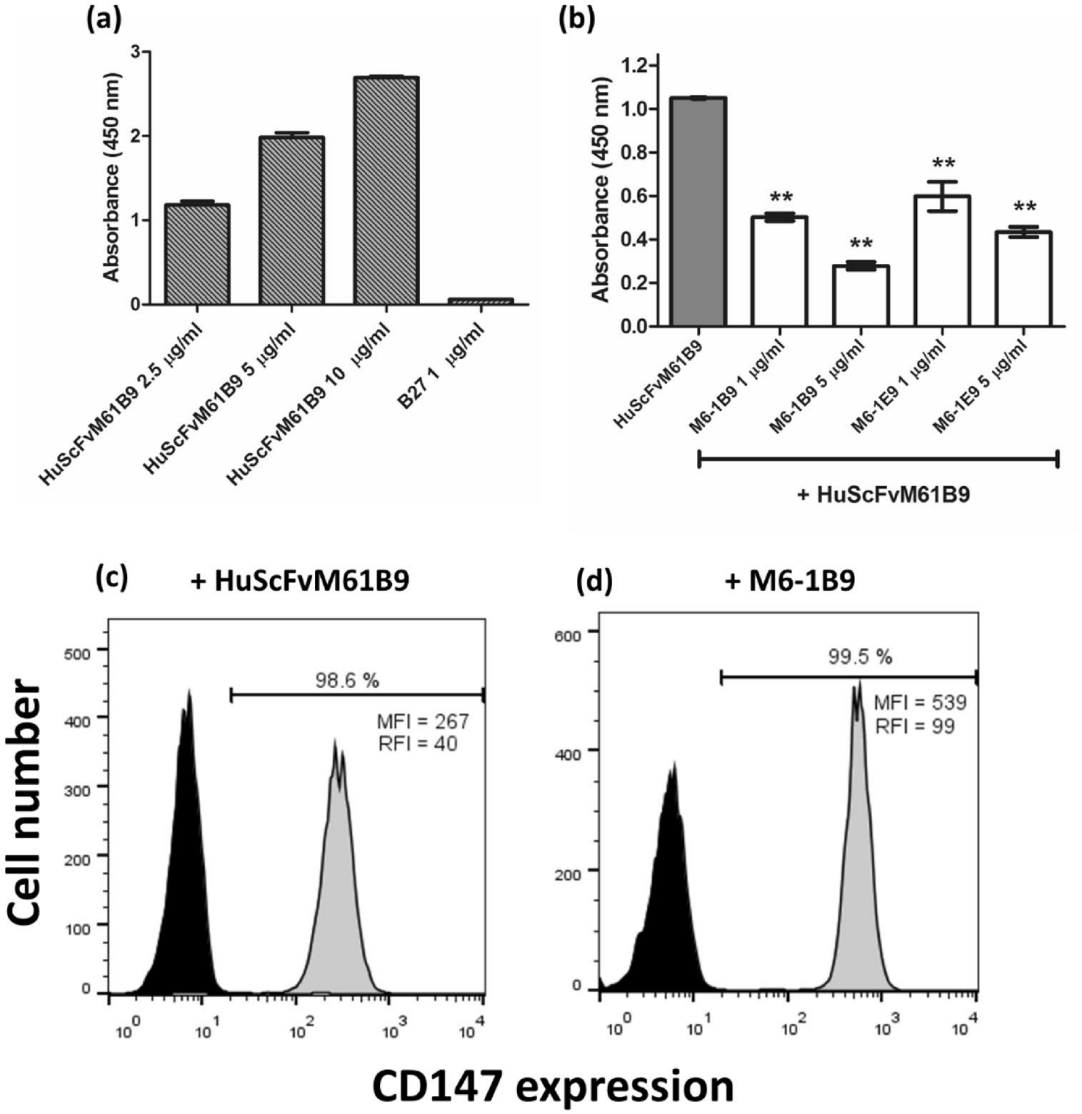
Immunoreactivity of HuScFvM61B9. (**a**) The microtiter plates were coated with CD147Rg, and HuScFvM61B9 was subsequently added at various concentrations to analyze the binding activity of HuScFvM61B9. HuScFvM61B9 was detected by HRP-conjugated anti-His-tag mAb. B27 was used as an irrelevant antibody control. (**b**) The ELISA wells were coated with CD147Rg, and mouse anti-CD147 mAbs were used to inhibit the binding of HuScFvM61B9 to the target. The signal was determined using HRP-conjugated anti-His-tag mAb, and the absorbance was measured using a microtiter plate reader. The data illustrate triplicate experiments and an error bar (mean ± SD), analyzed by a paired t-test (**p < 0.01). Immunofluorescence analysis of the reactivity of HuScFvM61B9 (**c**) and M6-1B9 (**d**) to CD147 on SupT1 cells. PE-conjugated anti-His tag antibody and Alexa Fluor 568 conjugated goat-anti-mouse IgG (H + L) were used as secondary antibodies. The percentage of positive cells is presented over the mean fluorescence intensity (MFI) and relative fluorescence intensity (RFI) values.

The binding activity of the purified HuScFvM61B9 to CD147 was investigated. The OD values at 450 nm were increased in a concentration-dependent manner (Fig. 8a). B27 was used as an irrelevant antibody control. This result suggested that purified HuScFvM61B9 was successfully bound to its target, CD147.

### Inhibition binding analysis of HuScFvM61B9 and anti-CD147 mAbs

Inhibition binding activity was determined using inhibition ELISA. The mouse anti-CD147 mAbs (M6-1B9 and M6-1E9) significantly inhibited the binding of purified HuScFvM61B9 to immobilized CD147 extracellular domain-human IgG Fc fusion protein (CD147Rg) in a concentration-dependent manner (Fig. 8b). These data demonstrated that HuScFvM61B9, M6-1B9, and M6-1E9 were bound to CD147 at the same or contiguous epitope.

### Reactivity of HuScFvM61B9 to CD147 on SupT1 cells

Flow cytometric analysis was performed to evaluate the functional activity of HuScFvM61B9 against CD147 on SupT1 cells. The HuScFvM61B9 was firmly bound to CD147 on the cell surface of SupT1 (Fig. 8c), comparable to that with its parental M6-1B9 mAb (Fig. 8d). However, the relative fluorescence index (RFI) of HuScFvM61B9 and M6-1B9 mAb compared with their conjugated controls was 40 and 99, respectively.

### Specific binding activity of HuScFvM61B9

**Figure 9:**
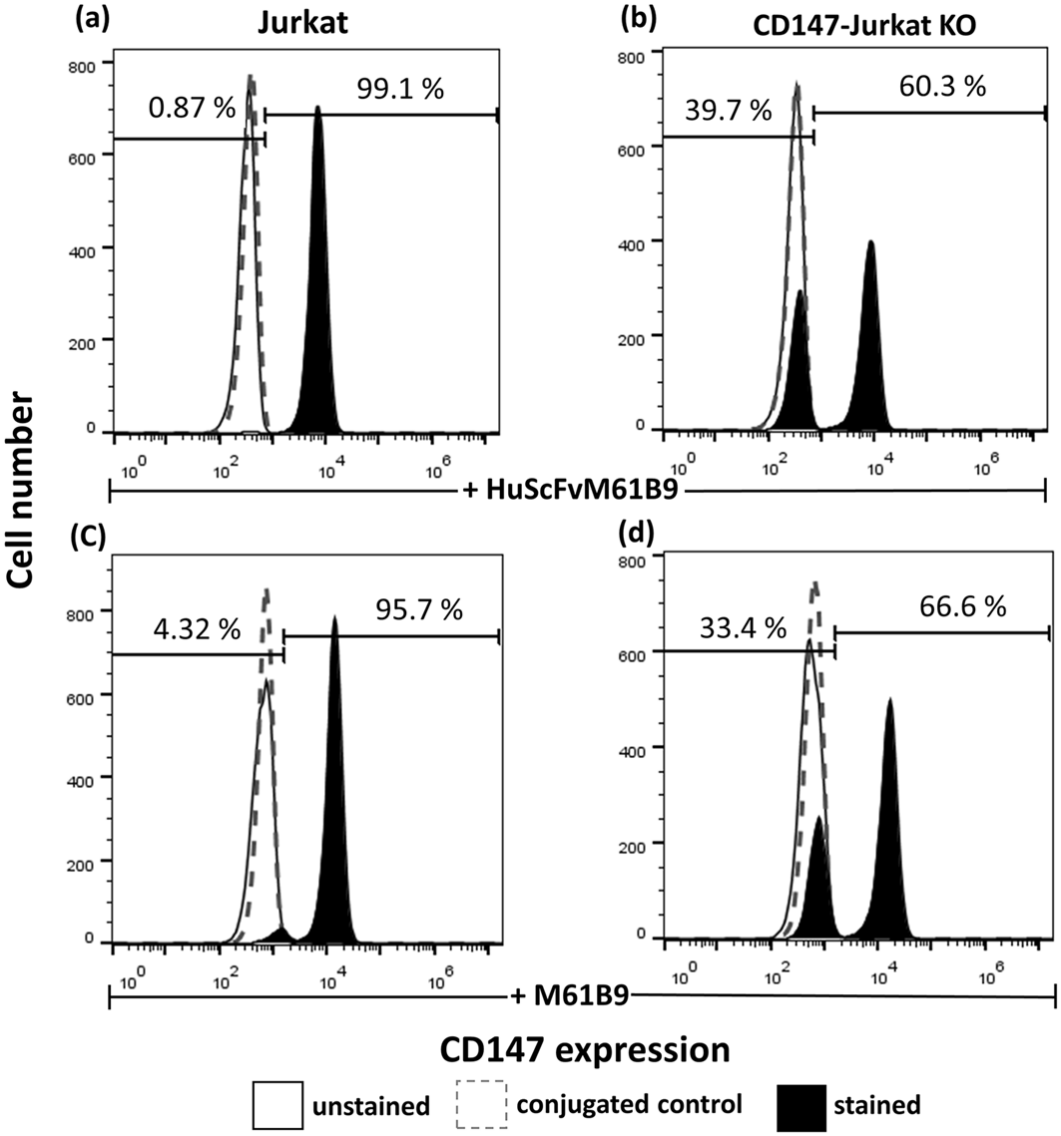
Specific binding activity of HuScFvM61B9. The Cas9 RNP delivery generated CD147 knockout Jurkat cells (CD147-Jurkat KO). Untransfected Jurkat and CD147-Jurkat KO cells were stained with HuScFvM61B9 (**a**,**b**) and M6-1B9 mAb (**c**,**d**), then followed by PE-conjugated anti-His tag antibody and Alexa Fluor 488 conjugated goat-anti-mouse IgG (H + L), respectively. Flow cytometric analysis was performed.

To determine the specific binding activity of HuScFvM61B9, the CD147 knockout Jurkat (CD147-Jurkat KO) was established. Jurkat cells were nucleofected with the ribonucleoprotein (RNP) complex using the sgRNA designed to target an exon 1 of the CD147 gene^34^. At day 11 post-nucleofection, the edited cell pool was collected to determine the CD147 expression using 40 μg/ml HuScFvM61B9 and 40 μg/ml M6-1B9 mAb. The flow cytometric analysis indicated approximately 39.7% and 33.4% of CD147-negative cells in CD147-Jurkat KO when stained with HuScFvM61B9 and M6-1B9, respectively (Fig. 9). In contrast, 99.1% and 95.7% of CD147-positive cells were shown in untransfected Jurkat cells when stained with HuScFvM61B9 and M6-1B9, respectively.

### HuScFvM61B9 inhibited anti-CD3 mAb-induced T cell proliferation

**Figure 10:**
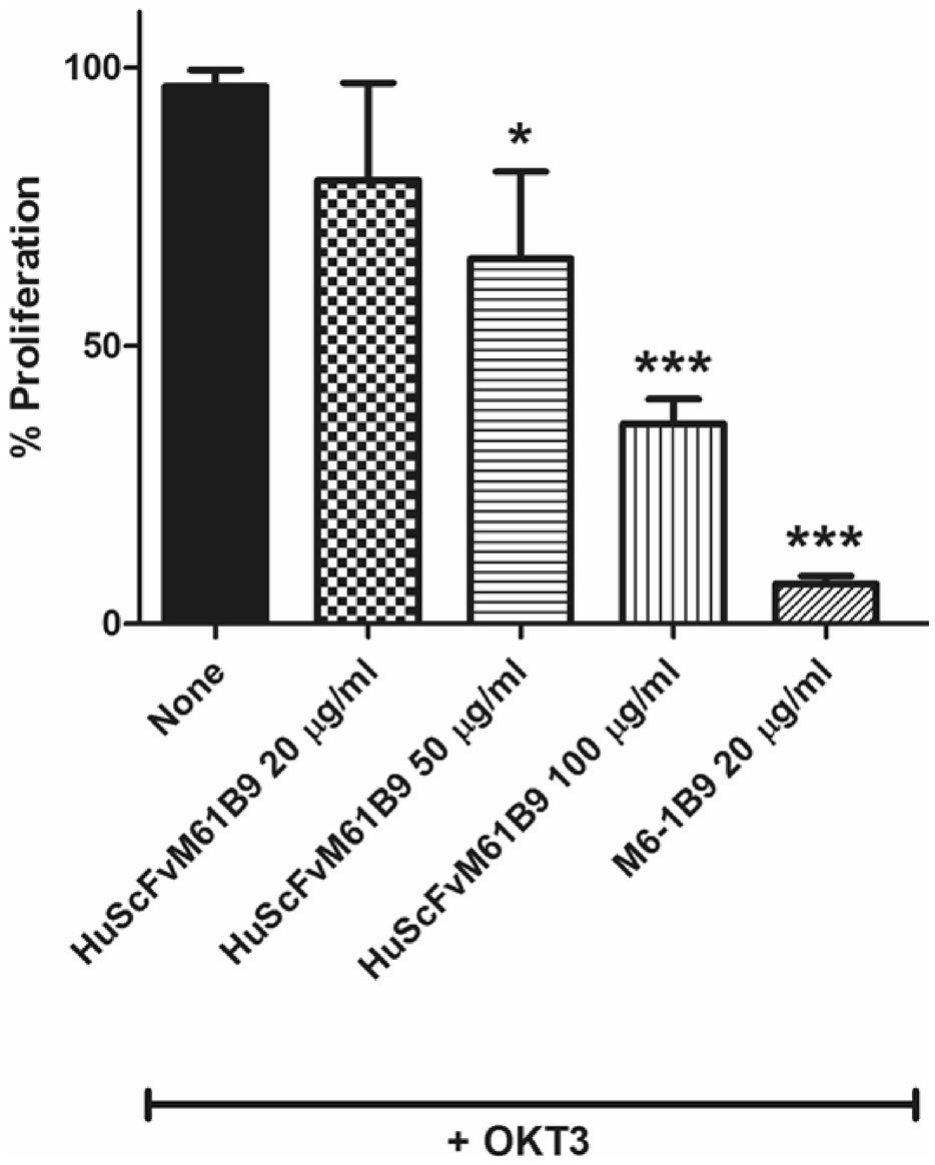
Inhibitory effects of HuScFvM61B9 on OKT3-induced T cell proliferation. PBMCs were stimulated with OKT3 in the presence of a various concentration of HuScFvM61B9. Cell proliferation was analyzed using flow cytometry by 5-carboxyfluorescein diacetate succinimidyl ester (CFSE) labeling. The data represented triplicate experiments and error bar (mean ± SD) performed by unpaired t-test (*p < 0.05, ***p < 0.001).

The parental M6-1B9 mAb and mouse ScFvM61B9 demonstrated their biological function on inhibiting anti-CD3 (OKT3)-mAb induced T-cell proliferation in previous reports^26,28^. Thus, the biological activity of HuScFvM61B9 was also assessed on anti-CD3 (OKT3)-mAb induced T-cell proliferation in this study. Peripheral blood mononuclear cells (PBMCs) from a healthy donor were activated with immobilized OKT3 in the presence or absence of HuScFvM61B9. The M6-1B9 mAb was used control. The results showed that HuScFvM61B9 inhibited the OKT3-induced T-cell proliferation as its parental, M6-1B9, in a dose-dependent manner (Fig. 10). However, the inhibitory effect of HuScFvM61B9 on OKT3-induced T-cell proliferation was lower than M6-1B9.

## Discussion

The T20 humanness scores for the heavy and light chains of HuScFvM61B9 increased over those of the parental sequence. As a weak correlation between the T20 score of 65 therapeutic antibodies and their available immunogenicity data^35^, our data suggested that the immunogenicity of HuScFvM61B9 was reduced and could potentially decrease the chance of HuScFvM61B9 immunogenicity in humans. Using the T20 humanness score, complicated and time-consuming in vitro experiments for immunogenicity prediction of HuScFvM61B9 were not required^35^.

Phage display combinatorial peptide libraries have been successfully and efficiently used to identify a wide variety of epitopes and mimotopes from a wide variety of mAbs^36,37^. Although a linear epitope can be mapped directly to the target protein, it must be combined with computer modelling to identify binding sites on the natural target. The amino acid sequences of phage clones 10, 12, and 14 were aligned with the human CD147 sequence and shared less than three continuous amino acid residues identical to the human CD147 sequence registered in GenBank. This indicated that these peptides were likely the mimotope of CD147. The EDLGS motif was an antigenic determinant, as it was not present in mouse CD147. These data suggested that M6-1B9 mAb recognized the epitope different from other anti-CD147 mAbs, including HAb18, which is used as a therapeutic antibody in hepatocellular carcinoma^16,17^. In addition, the M6-1B9 mAb recognized the EDLGS, which resides in the residues ^22^AAGTVFTTV*EDLGS*KILLTCSLNDSATEV^50^ in domain 1 of CD147; these residues have been suggested to play a critical role in MMP secretion and tumor invasion^17^.

Furthermore, the complex structure predicted by the ClusPro docking algorithm provided crucial residues for the antigen–antibody at the binding site. The analyzed RMSD was 0.758; by comparison, the Å values of mouse ScFvM61B9 and HuScFvM61B9 indicated a similar conformation between these two ScFvs. Interestingly, the predicted complex from ClusPro was similar to the structural coordinate generated by the Rosetta SnugDock protocol (data not shown).

The amino acids of each HuScFvM61B9 CDR interacting with the epitope on domain 1 of CD147 are listed in Fig. 1b. The significance of the EDLGS motif was elucidated by constructing the CD147 mutant, i.e., CD147Δ^32^DL^33^-BCCP. While HuScFvM61B9 and M6-1B9 specifically interacted with wild-type CD147, the immunoreactivity was absent with CD147Δ^32^DL^33^-BCCP. This phenomenon supports that the specificity of HuScFvM61B9 was retained for the EDLGS motif in domain 1 of CD147. Domain 1 of CD147 is crucial for MMP production, which leads to the degradation of the basement membrane and extracellular matrix and promotes tumor proliferation, invasion, and metastasis^15^. Taken together, HuScFvM61B9 would possess the necessary functional activities, including the ability to reduce MMPs, needed to ensure a feasible targeted cancer immunotherapy regimen.

When humanized ScFv is generated, the preservation of its affinity and biological function must be considered. The binding affinity of HuScFvM61B9 (7.97 ± 0.53 × 10^–8^) was lower than its parental mAb, M6-1B9 (3.11 ± 1.28 × 10^–9^). This phenomenon was similar to the previous report of anti-Her2 scFv^38^. Considering the biological function of HuScFvM61B9, the immunoreactivity of HuScFvM61B9 revealed that HuScFvM61B9 could specifically bind to CD147 at the same epitope as its parental mAb, M6-1B9 (Fig. 7b). In addition to M6-1B9, M6-1E9 inhibited HuScFvM61B9 from binding to its target because M6-1B9 and M6-1E9 bind to the same or contiguous epitope in domain 1 of CD147^26,39^. Moreover, HuScFvM61B9 is also bound to a leukemic cell line, SupT1, with low relative fluorescence intensity. CD147-Jurkat KO cells successfully confirmed the specific binding activity of HuScFvM61B9 and M6-1B9 by flow cytometry analysis (Fig. 9). The Cas9 RNP delivery was selected to generate the CD147-Jurkat KO because of its lower off-target mutation frequency than plasmid transfection with comparable on-target mutation frequency^40^. Moreover, the inhibitory effect of HuScFvM61B9 on OKT3-induced T cell proliferation was observed. Regarding the lower binding activity of HuScFvM61B9, its inhibitory effect was lower than M6-1B9 mAb (Fig. 10). Lateral movement of cellular membrane protein also possibly influences the different functional activity of HuScFvM61B9 and M6-1B9 mAb to CD147 on the cells, reflecting distinctive proliferation inhibition property.

Taken together, these results imply that a humanized ScFv targeting domain 1 of human CD147 (HuScFvM61B9) was successfully constructed and retained its immunoreactivity and functional activity of its parental M6-1B9 mAb. The development of HuScFvM61B9-Fc fusion (HuScFvFcM61B9) or fully humanized M6-1B9 IgG will be attempted in future research to improve the binding activity of monovalent ScFv.

## Materials and methods

### Epitope mapping via phage display random peptide library

Bio-panning of a 12-mer phage display random peptide library (SUT12) against the anti-CD147 mAb (clone M6-1B9) was performed as noted in previous research^36,41^. Briefly, three rounds of biopanning were performed by gradually reducing the amount of the M6-1B9 antibody, ranging from 10 to 5 and 2 µg for each consecutive round of affinity selection. After three rounds of biopanning, 16 individual phage clones were randomly chosen and amplified to assess their binding activity against the M6-1B9 mAb by phage ELISA, as previously reported^42^. The phagemid DNA from positive phage clones was prepared for nucleotide sequencing through automated DNA sequencing services using the -96gII primer (5′-CCC TCA TAG TTA GCG TAA CG-3′). The amino acid sequences were analyzed using SnapGene software.

### Humanization of ScFvM61B9 and property validation

The suitable frameworks in the variable domain of HuScFvM61B9 were assigned from parental ScFvM61B9 using the antibody humanization process of the BioLuminate 4.0 demo software (Schrödinger, LLC, USA)^27^. Upon submitting the amino acid sequence of ScFvM61B9 heavy and light chain variable domains, a PDB number 6N4Q crystal structure containing a mouse Fab template was selected for conforming to the possible structure. Subsequently, the human frameworks of PDB number 5HYS were automatically retrieved to substitute the frameworks of the modelled ScFvM61B9 structure to retain the canonical shape of antibody CDR loops. The structure comparison and RMSD calculation of the designed HuScFvM61B9 versus parental ScFvM61B9 were performed using UCSF Chimera 1.15 software. The CDR regions of the generated HuScFvM61B9 structure were deduced using the PyIgClassify Web server^43^. The humanness of HuScFvM61B9 was calculated using the T20 score analyzer tool^35^.

### Molecular model of HuScFvM61B9 against CD147 domain 1

The generated structure of HuScFvM61B9 from BioLuminate was submitted to the ClusPro 2.0 Web server along with the CD147 domain 1 extracted from PDB number 5X0T^44^. The antibody mode option was selected to automatically mask non-CDR regions in this in silico protein–protein docking process.

### Construction of plasmid expressing HuScFvM61B9

The amino acid sequence of modified ScFvM61B9 was reverse transcribed and optimized using the GenScript web service for proper expression in *E. coli*. The *HuScFvM61B9* coding sequence containing 5′ *Nhe*I and 3′ *Hind*III restriction sites was synthesized (GenScript, USA). The synthesized polynucleotide was subsequently digested with *Nhe*I and *Hind*III and cloned into the *Nhe*I and *Hind*III sites of the pET-21a plasmid vector to generate the pET-21a-*HuScFvM61B*9*(HIS6X)* plasmid.

### Expression and purification of HuScFvM61B9

The pET-21a-*HuScFvM61B9(HIS6X)* plasmid was transformed into competent *E. coli* Origami B (DE3) cells to produce a humanized single-chain variable fragment of M6-1B9 containing a His6x tag (HuScFvM61B9). A single colony was picked and grown in a 5 ml super broth medium starter culture overnight at 37 °C. Then, the culture was inoculated into a 500 mL SB medium containing 0.05% glucose and 100 µg/mL ampicillin at 37 °C until an OD_600_ of 0.8 was reached. Protein expression was induced by adding 50 µM IPTG, and this process continued for 16–18 h at 20 °C. The induced bacteria expressing HuScFvM61B9 were washed twice with PBS and lysed via three sonication times of 5 min each at 0.5 cycles with 80% amplitude on ice. The lysed bacteria were subjected to freeze–thaw cycling, followed by centrifugation at 4000*g* for 30 min at 4 °C. The cell lysate was collected, and HuScFvM61B9 was purified via affinity chromatography on a HisTrap HP column (GE Healthcare) using ÄKTA Prime plus. The collected fractions were analyzed via SDS-PAGE on a 15% gel with PageBlue staining to determine the purity of the HuScFvM61B9 proteins. The collected fractions were subjected to Western blot analysis. Proteins were separated by SDS-PAGE, transferred onto a PVDF membrane, and subsequently probed with an HRP-conjugated anti-His-tag antibody (BioLegend, 652504) at 1:3000 dilution. The reaction was developed using a chemiluminescent substrate detection system.

### Affinity determination of HuScFvM61B9 via biolayer interferometry

Biolayer interferometry was used to analyze the binding affinity of HuScFvM61B9 to human CD147-BCCP and compared to its parental mouse mAb, M6-1B9. The CD147-BCCP was in situ biotinylated in *E. coli* strain Origami B harboring pAK400cb-CD147-BCCP as described previously^27,45^. The CD147-BCCP was validated by Western immunoblotting using either M6-1B9 mAb and followed by HRP-conjugated anti-mouse Igs antibody or HRP-conjugated Streptavidin, and stored at − 20 °C for further analysis. Two hundred microliters of 2 mg/ml crude CD147-BCCP were immobilized on Streptavidin biosensor tip (Sartorius FortéBio) and then placed to buffer (0.05% Tween-20 in PBS) to generate the baseline. To determine the association signal, the tips were subsequently placed into either mouse anti-CD147 mAb clone M6-1B9 or HuScFvM61B9 at 20, 10, and 5 μg/ml. After that, the tips were placed into the buffer to generate the dissociation signal. The constants of association (k_a_) and dissociation (k_d_) were analyzed, and the K_D_ was calculated via the same formular by BlitZPro 1 program.

### Specificity of HuScFvM61B9 against the wild-type CD147 and mutant CD147

To assess the specificity of HuScFvM61B9 by Western immunoblotting, wild-type CD147-BCCP (CD147WT-BCCP) and mutant CD147-BCCP (CD147Δ^32^DL^33^-BCCP) were generated. Briefly, deletion of ^32^DL^33^ in E*DL*GS of pAK400cb-CD147-BCCP was performed using QuickChange Lightning Multi Site-Directed Mutagenesis kit (Agilent) following the manufacturer’s instruction. Mutated primer DNA sequences are listed as follows.

- Fw_del32-33 5′-GCA CAG TCT TCA CTA CCG TAG AAG GCT CCA AGA TA-3′.
- Rv_del32-33 5′-TAT CTT GGA GCC TTC TAC GGT AGT GAA GAC TGT GC-3′.

The pAK400cb-CD147-BCCP-del32DL33 was subsequently transformed into competent *E. coli* DH5α, and the clones were selected on Luria–Bertani (LB) agar containing 25 μg/ml of chloramphenicol. The plasmid from transformed *E.* coli DH5α was prepared by QIAGEN Miniprep kit (QIAGEN) and sequenced. The corrected pAK400cb-CD147-BCCP-del32DL33 was transformed to *E. coli* strain Origami B to produce CD147Δ^32^DL^33^-BCCP, and validated as described previously^28,45^. Crude lysate protein at 1:2 dilution of both CD147WT-BCCP and CD147Δ^32^DL^33^-BCCP were subsequently separated by SDS-PAGE, transferred onto a PVDF membrane, and subsequently probed with HuScFvM61B9 (1 μg/ml) followed by HRP-conjugated anti-His-tag mAb at 1:1000 dilution, M6-1B9 mAb (0.5 μg/ml) followed by HRP-conjugated anti-mouse Igs (Dako, P0260) at 1:2000 dilution and HRP-conjugated Streptavidin (Seracare, 5270-0029) at 1:500 dilution. The reaction was developed using a chemiluminescent substrate detection system.

### Binding assay of HuScFvM61B9 by indirect ELISA

To investigate the binding activity of HuScFvM61B9 to CD147, the indirect ELISA was performed. The microtiter plates were immobilized with 50 µL of 10 µg/mL CD147Rg and incubated overnight at 4 °C in a moist chamber. The other steps were performed at ambient temperature in a moist chamber. The coated wells were washed three times with a washing buffer (0.05% Tween 20 in PBS), followed by blocking with 2% skim milk in PBS for 1 h. After washing, purified HuScFvM61B9 protein was added at various concentrations to the wells and incubated for 1 h. After washing three times, 50 µL of HRP-conjugated anti-His-tag mAb at 1:5000 dilution was added and incubated for 1 h. The reaction was developed using a TMB substrate, and then the reaction was ceased with 1 N HCl. Absorbance at 450 nm was measured using an ELISA reader.

### Inhibition binding analysis of HuScFvM61B9 with anti-CD147 mAbs via inhibition ELISA

Inhibition binding activity of purified HuScFvM61B9 was assessed using the inhibition ELISA. Fifty microliters of 10 µg/mL CD147Rg were immobilized on a microtiter plate and incubated overnight at 4 °C in a moist chamber. The other steps were performed at ambient temperature in a moist chamber. Non-specific protein binding was blocked using 2% skim milk in PBS. Fifty microliters of 1 and 5 µg/mL mouse anti-CD147 mAb (M6-1B9 and M6-1E9) were added and incubated for 1 h. The microtiter plate was washed three times with 0.05% Tween20 in PBS and 50 μL of 10 µg/mL purified HuScFvM61B9 was added to the well. After 1 h of incubation, the microtiter plate was washed three times with 0.05% Tween20 in PBS. HRP-conjugated anti-His-tag mAb at 1:5000 dilution was subsequently added to the well and incubated for 1 h. TMB substrate was then added to develop the reaction. The absorbance at 450 nm was measured using a microplate reader after adding 1 N HCl.

### Immunofluorescence analysis of the reactivity of HuScFvM61B9 to CD147 on SupT1 cells

Flow cytometric analysis was performed to evaluate the functional reactivity of HuScFvM61B9 against CD147 on a human T-cell lymphoblastic lymphoma cell line, SupT1, compared to its parental M6-1B9 mAb^25^. SupT1 cells were washed three times with a FACS buffer and adjusted to 10^7^ cells/mL with 10% human AB serum for 30 min on ice to block the Fc receptor. Fifty microliters of 10 µg/mL HuScFvM61B9 or M6-1B9 mAb was added into 50 μL of blocked cells and incubated on ice for 30 min. The cells were washed twice with 1% FBS in PBS. After washing, a PE-conjugated anti-His-tag antibody (BioLegend, 362603) at 1:20 dilution or conjugated anti-mouse Igs was added and incubated on ice for another 30 min. Finally, the cells were washed three times and fixed with 1% paraformaldehyde in PBS. The fluorescence intensity of the stained cells was analyzed using a BD FACSCelesta instrument and FlowJo software.

### Generation of CD147 knockout Jurkat cells for analysis the specificity of HuScFvM61B9

For the generation of CD147 knockout Jurkat cells, the sgRNA targeting CD147 molecule^34^ was generated using the GeneArt Precision gRNA Synthesis kit (Invitrogen, A29377). The RNP complex was prepared by mixing 150 μg/mL of SpCas9 (Integrated DNA Technologies) and 90 μg/mL of sgRNA at a 1:3 molar ratio, then incubated for 10 min at room temperature. 1 × 10^6^ Jurkat cells were nucleofected with RNP using the Cell Line Nucleofector Solution V (Lonza, VCA-1003) and the program X-005. On day 11, the edited cell pool (CD147 knockout Jurkat cells) was used to analyze the specificity of HuScFvM61B9 by immunofluorescence analysis using the protocol described above except 40 μg/ml concentration was used. Untransfected Jurkat cells were used as a positive control.

### Effect of HuScFvM61B9 on T-cell proliferation in PBMC after anti-CD3 antibody activation

T cell proliferation was assessed using 5-carboxyfluorescein diacetate succinimidyl ester (CFSE) labeling. PBMCs were washed with PBS 3 times and adjusted to 10^7^ PBMCs/ml with PBS. CFSE (Sigma-Aldrich) at a final concentration of 1 μM was added to PBMCs for 10 min at 37 °C. Excess CFSE was quenched with cold 10% FBS-RPMI. CFSE-labelled PBMCs were washed twice with RPMI. To determine the effect of HuScFvM61B9 on T-cell proliferation in PBMC after anti-CD3 activation, triplicate aliquots of 10^5^ CFSE-labeled PBMCs were cultured with immobilized CD3 mAb OKT3 (12.5 ng/mL) with various concentrations of purified HuScFvM61B9. Mouse M6-1B9 mAb was used as a control. The culture was incubated for 5 days in a 5% CO_2_ incubator at 37 °C. Cells from each treatment were then washed twice with PBS, fixed with 1% formaldehyde in PBS and analyzed using BD Accuri C6 flow cytometer and FlowJo software.

### Ethical declarations

The study protocol was approved by the Ethics Committee, Faculty of Associated Medical Sciences, Chiang Mai University, Thailand (AMSEC-64EX-016).
